# PA-X is an avian virulence factor in H9N2 avian influenza virus

**DOI:** 10.1099/jgv.0.001531

**Published:** 2021-02-05

**Authors:** Anabel L. Clements, Thomas P. Peacock, Joshua E. Sealy, Hui Min Lee, Saira Hussain, Jean-Remy Sadeyen, Holly Shelton, Paul Digard, Munir Iqbal

**Affiliations:** ^1^​ The Pirbright Institute, Pirbright, Woking, GU24 0NF, UK; ^2^​ The Roslin Institute and Royal (Dick) School of Veterinary Studies, University of Edinburgh, Edinburgh, EH25 9RG, UK; ^3^​ Department of Infectious Diseases, Imperial College London, W2 1PG, UK; ^†^​ Present address: The Francis Crick Institute, London, NW1 1AT, UK

**Keywords:** accessory protein, avian influenza, Influenza, PA-X, poultry, virulence factor

## Abstract

Influenza A viruses encode several accessory proteins that have host- and strain-specific effects on virulence and replication. The accessory protein PA-X is expressed due to a ribosomal frameshift during translation of the PA gene. Depending on the particular combination of virus strain and host species, PA-X has been described as either acting to reduce or increase virulence and/or virus replication. In this study, we set out to investigate the role PA-X plays in H9N2 avian influenza viruses, focusing on the natural avian host, chickens. We found that the G1 lineage A/chicken/Pakistan/UDL-01/2008 (H9N2) PA-X induced robust host shutoff in both mammalian and avian cells and increased virus replication in mammalian, but not avian cells. We further showed that PA-X affected embryonic lethality *in ovo* and led to more rapid viral shedding and widespread organ dissemination *in vivo* in chickens. Overall, we conclude PA-X may act as a virulence factor for H9N2 viruses in chickens, allowing faster replication and wider organ tropism.

## Introduction

Influenza A viruses (IAV) have segmented negative-sense RNA genomes encoding 10 core proteins and a variable number of strain-specific accessory proteins [1, 2]. Due to its small genome size and nuclear replication, IAV has evolved a number of ways to increase its protein coding capacity including the use of splice variants (M2, NEP and the more recently discovered M42, PB2-S1 and NS3 proteins), encoding multiple open reading frames (ORFs), both nested and overlapping on a single gene segment (e.g. PB1-F2, PB1-N40, NA43) and ribosomal frameshifts leading to multi-ORF fusion proteins (PA-X) [3–8]

Influenza A virus segment three primarily encodes the polymerase acidic protein (PA). PA is an integral part of the influenza virus RNA-dependent RNA polymerase (RdRp) and contains two functional domains, an N-terminal endonuclease (endo) domain, responsible for cleaving host capped RNAs used to prime viral transcription, and a C-terminal domain, associated with the core of the RdRp [9]. The 3′-boundary of PA endo domain coding sequence contains a conserved ribosomal frameshift site where a rare arginine codon facilitates ribosomal stalling, which is followed (at low level) by ribosomal slippage into a +1 open reading frame (ORF), facilitated by an upstream UUU codon that allows tRNA realignment [10, 11]. The resulting protein, PA-X, is a fusion between the first 191 amino acids of PA (the endo domain) and up to 61 amino acids translated from the X-ORF in the +1 reading frame. PA-X has been shown to mediate degradation of host cell mRNAs and disruption of host mRNA processing, leading to host cell shutoff and a dampened innate immune response [11–17].

H9N2 avian influenza viruses are enzootic through much of Asia, the Middle East and Africa [18]. H9N2 viruses cause massive economic damage due to their impact on poultry production systems, causing moderate morbidity and mortality, especially in the context of viral or bacterial coinfection. Additionally, H9N2 viruses pose a direct zoonotic threat to humans and are considered viruses with pandemic potential [18, 19]. As well as being zoonotic threats in their own right, H9N2 viruses have contributed polymerase genes to multiple zoonotic avian influenza viruses, including epidemic H7N9 [20].

Several studies have investigated the role of PA-X on virulence and replication of avian influenza viruses in mammalian and avian hosts. There is little consensus between these studies and there appears to be virus strain- and host species-specific differences in whether PA-X expression increases or decreases replication, virulence and transmissibility of IAV [11, 15, 21–29]. In H9N2 viruses, expression of full length PA-X has been shown to be a virulence factor in mammalian infection systems [21, 23]. However, it is unclear what role PA-X has in these viruses in their natural chicken hosts.

In this study we set out to investigate the role of PA-X in a contemporary G1-lineage H9N2 virus, typical of viruses circulating in the Middle East and South Asia [18]. We found that the H9N2 virus expressed a PA-X capable of robust host shutoff which correlated with PA-X expression. Removing PA-X expression decreased viral replication in mammalian, but not avian cell culture systems, although it did reduce embryonic lethality *in ovo. In vivo*, in the natural chicken host, ablation of PA-X expression led to delayed shedding and restricted viral dissemination. Overall, our results suggest PA-X may be an H9N2 virulence factor in the natural avian host, allowing faster replication and increased visceral organ tropism.

## Methods

### Ethics statement

All animal experiments were carried out in strict accordance with the European and United Kingdom Home Office Regulations and the Animal (Scientific Procedures) Act 1986 Amendment regulation 2012, under the authority of a United Kingdom Home Office Licence (Project Licence Numbers: P68D44CF4 X and PPL3002952).

### Cells

Madin-Darby canine kidney (MDCK) cells, human embryonic kidney (HEK) 293 T cells and chicken DF-1 cells were maintained in Dulbecco’s Modified Eagle Medium (DMEM; Sigma) supplemented with 10 % FBS and 100 U ml^−1^ Penicillin-Streptomycin (complete DMEM). All cells were maintained at 37 °C, 5 % CO_2_.

Primary chicken kidney (CK) cells were generated as described elsewhere [30]. Briefly, kidneys from 3 week-old SPF Rhode Island Red birds were mechanically shredded, washed, trypsinised, and then filtered. Cells were resuspended in CK growth media (EMEM +0.6 % BSA, 10 % v/v tryptose phosphate broth, 300 U ml^−1^ penicillin/streptomycin), plated and maintained at 37 °C, 5 % CO2.

### Viruses

All work in this study was performed with the reverse genetics derived H9N2 virus, chicken/Pakistan/UDL-01/2008 (UDL-01) [31]. UDL-01 virus segments were expressed in bi-directional reverse genetics PHW2000 plasmids [32]. Mutant PA segments were generated by site directed mutagenesis. PR8 WT and FS segment three were previously described [11].

Viruses were rescued as described elsewhere [32]. Briefly, 250 ng of each segment plasmid were co-transfected into 293Ts plated in six well plates using lipofectamine 2000. At 16 h post-transfection, media was changed to reverse genetics media (DMEM +2 mM glutamine, 100 U ml^−1^ penicillin, 100 U ml^−1^ streptomycin, 0.14 % BSA, 5 µg ml^−1^ TPCK-treated trypsin). Following a 48 h incubation at 37 °C, 5 % CO_2_, supernatants were collected and inoculated into embryonated hens’ eggs for subsequent harvest of virus infected allantoic fluid. Virus segment three sequences were confirmed by RT-PCR and Sanger sequencing.

### *In vitro* translation and autoradiography

For *in vitro* translations to visualise radiolabelled proteins, the TnT Coupled Reticulocyte Lysate system (Promega; #L4610) was utilised as per the manufacturers’ instructions using 200 ng of T7 promoter containing PHW2000 plasmid DNA. Reactions were incubated at 37 °C for 90 min then denatured in 50 µl protein loading buffer and analysed on a 15 % SDS-PAGE, to allow greatest and autoradiography. X-ray films were developed using a Konica SRX-101A X-ograph film processor as per the manufacturers’ instructions.

### Host cell shutoff assays

The β-gal shutoff reporter assays were performed as described elsewhere [11]. Briefly, 293T or DF-1 cells were co-transfected with expression plasmids for the influenza segment 3 (PA) and β-galactosidase (β-gal) reporter. Then 48 h later, cells were lysed with 100 µl of 1 x Reporter lysis buffer (Promega). β-gal expression was assayed using the β-galactosidase enzyme assay system (Promega). A Promega GloMax Multi Detection unit was used to measure absorbance at 420 nm. Western blotting was performed according to standard protocols. PA was detected using a rabbit polyclonal raised to bacterially-expressed PA [33], while tubulin was detected with rat (Abcam; YL1/2) or rabbit (Abcam; ab15246) monoclonal antibodies as a loading control.

For the shutoff activity assays using live virus, MDCKs were infected with virus at a high MOI of 5. At 7.5 h post-infection, cells were washed and the medium changed to 1 ml of complete DMEM containing 10 µg ml^−1^ of Puromycin dihydrochloride from *Streptomyces alboniger* for 30 min. Cells were washed and lysed in protein loading buffer and run on an SDS-PAGE and were then Western blotted, probing for puromycin with a mouse monoclonal antibody to puromycin (Millipore; MABE343). Puromycylated protein synthesis was quantified in the region of the gel between 45 kDa and 80 kDa. Protein quantification following Western blot was measured by densitometry using ImageJ analysis software.

### Mini-replicon assays

The 293T or DF-1 cells were transfected with plasmids encoding PB2, PB1, PA and NP, along with a firefly luciferase vRNA-like reporter under a cell-type specific polI promoter (human for 293T, chicken for DF1). The following concentration of plasmid were used for 293Ts, PB2- 80 ng, PB1- 80 ng, PA- 20 ng, NP- 160 ng, pPol I Luc- 800 ng, these amounts were doubled for DF1s. After 48 h, media was removed, and cells were lysed in 100 µl of 1 x Passive Lysis Buffer (Promega). A Promega GloMax Multi Detection unit was used to measure luciferase activity following the manufacturer’s instructions.

### Virus replication assays

MDCK and CK cells were infected at a low MOI of 0.01 for 1 h in serum-free DMEM, after which media was replaced with DMEM, 2 µg ml^−1^ tosyl phenylalanyl chloromethyl ketone (TPCK)-treated trypsin (MDCK cells) or Eagle’s minimum essential medium (EMEM), 7 % bovine serum albumin [BSA], and 10 % tryptose phosphate broth (CKs). Time points were harvested in triplicate at 4-, 8-, 12-, 24-, 48- and 72 h post-infection. Virus titres were determined by plaque assay on MDCK cells.

The 10 day old embryonated hens’ eggs (VALO breed) were inoculated with 100 p.f.u. of diluted virus into the allantoic cavity. Eggs were incubated for 4–72 h and culled via the schedule one method of refrigeration at 4 °C for a minimum of 6 h. Five eggs were used per virus per time point. Harvested allantoic fluid from each egg was collected and virus titres were assessed by plaque assay on MDCK cells.

For the egg mortality rates experiment a 10-fold serial dilution of each virus (10 000 to 10 p.f.u.) was made as used to infect five embryonated eggs per virus per dilution. Embryos were candled twice daily throughout the study period to check for embryo viability (up to 84 h post-infection). If eggs reached a predetermined end point at candling, they were deemed to be dead and the eggs chilled to ensure death before disposal. Markers of the end point included, a lack of movement of the embryo, disruption of blood vessels within the egg and/or signs of haemorrhage. If the embryos survived until the experimental end (84 h post-infection) they were culled via a schedule one method and samples of allantoic fluid were collected to determine presence of virus via immunostaining for viral NP protein. Any eggs without positive detection of viral NP were removed from the study.

### Virus infection, transmission and clinical outcome *in vivo*

*In vivo* studies were performed with 3 week-old White Leghorn birds (VALO breed). Prior to the start of the experiments, birds were swabbed and bled to confirm they were naïve to the virus. All infection experiments were performed in self-contained BioFlex B50 Rigid Body Poultry isolators (Bell Isolation Systems) at negative pressure. Then 10 birds per group were directly inoculated with 10^4^ p.f.u. of virus via the intranasal route. Mock infected birds were inoculated with sterile PBS as an alternative. One day post-inoculation eight naïve contact birds were introduced into each isolator to determine viral transmissibility.

Throughout the experiment, birds were swabbed in the buccal and cloacal cavities (on day 1–8, 10 and 14 post-infection). Swabs were collected into 1 ml of virus transport media (WHO standard). Swabs were soaked in media and vortexed for 10 s before centrifugation. Viral titres in swabs were determined via plaque assay.

At day 2 post-infection, three birds per group (directly infected, contact and mock infected) were euthanised via overdose of pentobarbital (at least 1 ml). A panel of tissues were collected and stored in RNA later at −80 °C until further processing for qRT-PCR of virus gene copies and cytokines. Birds were observed twice daily by members of animal services and whilst procedures were carried out for the presence of clinical signs of infection. On day 14 post-infection, all remaining birds were culled via overdose of pentobarbital or cervical dislocation.

### RNA extraction and RT-PCR from chicken tissues

The 30 mg of tissue collected in RNA later was mixed with 750 µl of Trizol. Tissues were homogenised using the Retsch MM 300 Bead Mill system (20 Hz, 4 min). Then 200 µl of chloroform was added per tube, shaken vigorously and incubated for 5 min at room temperature. Samples were centrifuged (9 200 ***g***, 30 min, 4 °C) and the top aqueous phase containing total RNA was added to a new microcentrifuge tube, subsequent RNA extraction was then carried out using the QIAGEN RNeasy mini kit following manufacturers’ instructions.

Next 100 ng of RNA extracted from tissue samples was used for qRT-PCR. All qRT-PCR was completed using the Superscript III platinum One-step qRT-PCR kit (Life Technologies) following manufacturer’s instructions for reaction set up. Cycling conditions were as follows: i) 5 min hold step at 50 °C, ii) a 2 min hold step at 95 °C, and 40 cycles of iii) 3 s at 95 °C and iv) 30 s annealing and extension at 60 °C. Cycle threshold (CT) values were obtained using 7500 software v2.3. Mean CT values were calculated from triplicate data. Negative controls were included within each plate to determine any unspecific amplification or contamination. Within viral M segment qRT-PCR an M segment RNA standard curve was completed alongside the samples to quantify the amount of M gene RNA within the sample from the CT value. T7 RNA polymerase-derived transcripts from UDL-01 segment seven were used for the preparation of the standard curve.

Within the cytokine qRT-PCRs, three housekeeping genes were included per sample (RPLPO-1, RPL13 and 28S rRNA) that had been previously determined to be stable in a broad range of tissues. Briefly, the geNorm algorithm (Vandesompele *et al.*, 2002) was adopted to calculate the stability for each reference gene and the optimal reference gene number from raw Cq values of candidate reference genes using qbase +real time qPCR software version 3.0 (Biogazelle).

### Statistical analysis

All statistical analysis was carried out using GraphPad Prism 6/7 software. Parametric vs non-parametric distribution of data was assessed prior to deciding on the statistical test to use. Correlation coefficients were calculated using Spearman’s Rank correlation coefficient.

## Results

### Generation of H9N2 viruses with altered PA-X expression

**Fig. 1.**
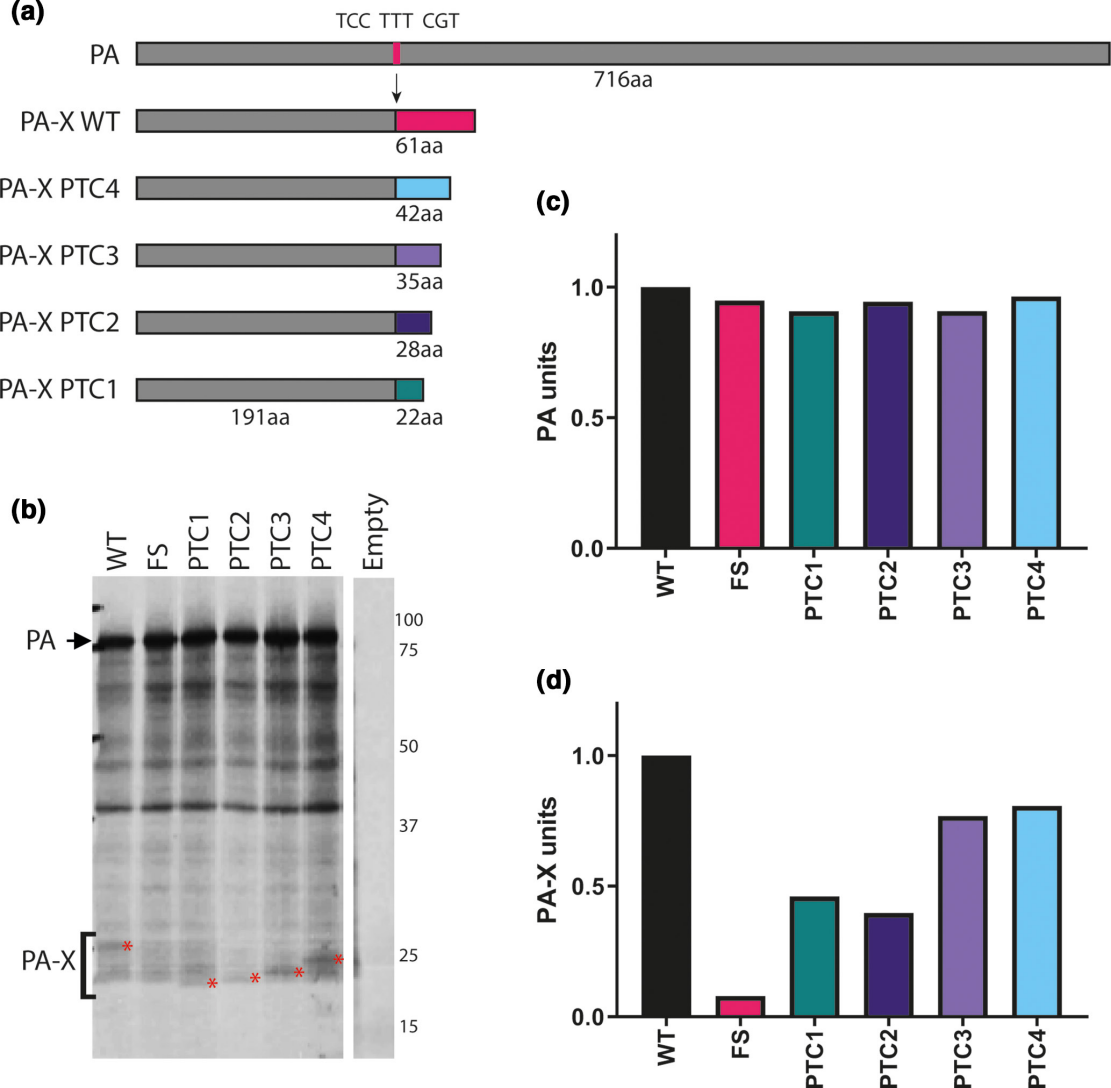
Generation and validation of H9N2 viruses with mutant PA-X proteins. A panel of mutations were made within UDL-01 segment three that altered PA-X expression. (a) Location of mutations within frameshift site and X-ORF. Dark grey rectangle represents PA, light grey rectangle represents X-ORF. Pink line represents location of the frameshift site (FS), coloured lines represents location of PTC mutations. (b) Coupled *in vitro* transcription-translation reactions radiolabelled with ^35^S-methionine were carried out using the TnT rabbit reticulocyte lysate system and protein products analysed using a 15 % SDS-PAGE gel and autoradiography. Red asterisks indicate PA-X polypeptides. PA is marked via a black arrow. (c) Quantification of the AUC of the densitometry analysis of the PA band using ImageJ analysis software. (d) Quantification of the AUC of the densitometry analysis of the PA-X band using ImageJ analysis software in the area indicated by the bracket and compared to local background.

To investigate the role of PA-X in an H9N2 background we generated a panel of mutants in the background of a contemporary G1-lineage H9N2 virus UDL-01, typical of viruses still circulating in South Asia and the Middle East [34, 35]. Mutants were generated (in cDNA copies of segment three cloned into a reverse genetics plasmid) using a previously validated approach, with the frameshift site mutated in such a way that ribosomal slippage should be inhibited (FS); additionally, a panel of truncated PA-X mutants with premature termination codons (PTC) spaced throughout the X-ORF, were made (Table 1, Fig. 1a) [11, 25]. All mutations were synonymous in the coding sequence of PA.

**Table 1.** Summary of PA-X mutants made in this study

Mutation name	Original nt	Mutated nt	nt position(s)	PA-X amino acid change
**FS**	TCC TTT CGT	AGC TTC AGA	568,569,573,574,576	
**PTC1**	C	A	621	I207STOP
**PTC2**	C, C, G	A, G, A	634 636 642	R212STOP, L214STOP
**PTC3**	T	A	678	L226STOP
**PTC4**	G	A	699	V233STOP

To confirm ablation of PA-X expression and/or validate the truncations, *in vitro* transcription/translation assays in rabbit reticulocyte lysate were carried out directly from the plasmids. Comparable levels of PA expression were seen across every construct, confirming the PA-X mutations did not affect PA expression (Fig. 1b, c). As expected [11, 15, 25], PA-X expression was visible as a minor polypeptide species migrating above the 25 kDa marker, whose abundance was drastically reduced in the FS mutant (Fig. 1b red asterisks, D). Upon X-ORF truncation, a ladder effect where the size of PA-X was progressively decreased by the PTC mutations could be seen, while both PTC1 and PTC2 also showed a reduction of PA-X expression. Overall, these results indicate that a previously used strategy for altering PA-X expression is also successful in this H9N2 virus background.

### A PA-X frameshift mutation abrogates host cell shutoff activity

**Fig. 2.**
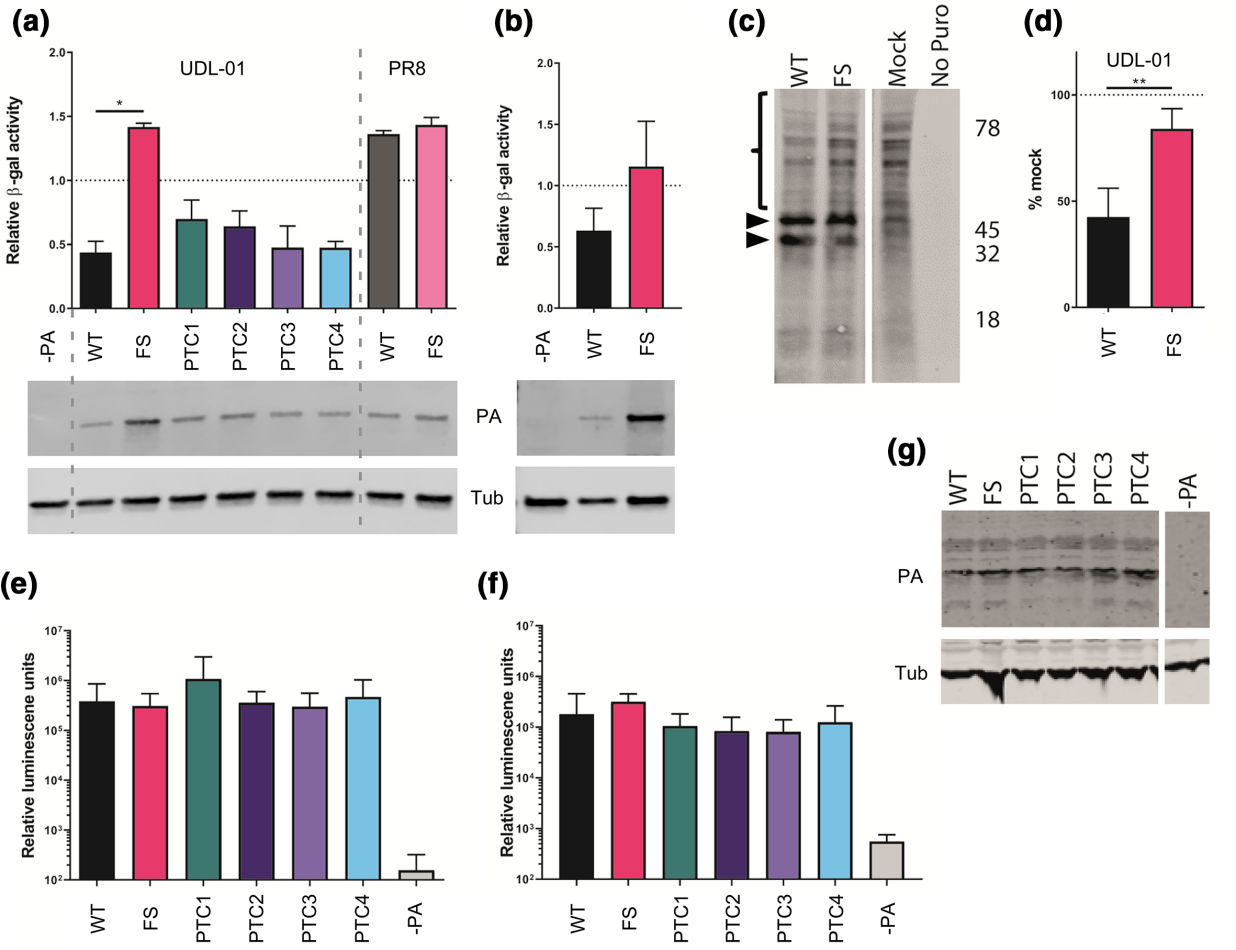
Ablation of PA-X expression leads to a loss of host cell shutoff but has no effect on virus polymerase activity. (a) The 293 T cells or (b) DF-1 cells were transfected with a β-gal reporter plasmid alongside the indicated segment three expression plasmids with or without PA-X mutations. Then 48 h post-transfection cells were lysed and levels of β-gal enzymatic activity assessed by colorimetric assay. Results were normalised to an empty vector control. Graphs represent the average ±SD of three independent experiments. Statistical significance was determined by one-way ANOVA with multiple comparisons (a) or unpaired *T*-test (b). Lower panels; cell lysates (collected separately) were analysed by Western blotting for PA and (as a loading control), α-tubulin (Tub). (c, d) MDCK cells were infected with a high MOI [5] using viruses expressing PA-X (WT) or with PA-X expression removed (FS). Seven hours post-infection cells were pulsed with puromycin for 30 min and then lysed and samples run on SDS-PAGE gels. Membranes were probed for presence of puromycin. (c) Representative Western blot with the area quantified highlighted by a bracket. The approximate position of molecular mass markers (kDa) is also indicated. Both blot halves originate from a single membrane processed as a whole but with unwanted lanes removed during figure preparation. (d) Quantification of the densitometry of the highlighted area (above 47 kDa) performed using ImageJ analysis software. Graph represents the average of three independent experiments ±SD. Each data group is normalised to mock levels of protein synthesis. Statistics determined by unpaired *T*-test. (e) The 293 T cells or (f) DF-1 cells were transfected with the components of the polymerase complex (PB1, PB2, PA and NP) plus a vRNA mimic encoding luciferase. Then 48 h post-transfection cells were lysed and luciferase levels measured. Data are the average of three independent experiments ±SD. Statistics were determined by Kruskal Wallis test with multiple comparisons. (g) PA and tubulin expression levels were determined via Western blot analysis of 293 T cell lysates from part (e) Blot halves originate from a single membrane processed intact but with unwanted lanes removed during figure preparation. *P* values for statistics throughout: *, 0.05 ≥P >0.01; **, 0.01 ≥P >0.001.

PA-X plays a key role in IAV host cell shutoff, therefore the ability of the H9N2 PA-X variants to repress cellular gene expression was tested using transfected segment three plasmids in β-galactosidase (β-gal) reporter assays. HEK 293Ts or DF-1 cells were transfected with the β-gal plasmid along with segment three plasmids or an empty vector control, β-gal accumulation was measured by enzyme assays 48 h later and normalised to empty vector. UDL-01 WT PA-X but not the A/Puerto Rico/8/34 (PR8) PA-X showed robust repression of β-gal expression in both cell types, and introduction of the FS mutant ablated the UDL-01 activity (Fig. 2a, b), indicating that the shutoff activity of segment three is dependent on PA-X expression but varies according to IAV strain, as previously shown [11, 17, 25]. When shut-off activity of the UDL-01 PTC mutants was tested in 293 T cells, PTC1 and PTC2 showed a minor and non-statistically significant reduction in host shutoff activity compared to UDL-01 WT while PTC3 and PTC4 had no apparent effect. Western blot analysis of PA expression confirmed similar expression levels of the various PA polypeptides, with the exception of the UDL-01 FS mutant, which accumulated to higher levels in both 293T and DF1 cells (Fig. 2a, lower panels). The increased expression of the FS mutant may reflect removal of the auto-repressive PA-X function from the segment, as previously noted [11]. Thus UDL-01 encodes an active PA-X polypeptide, whose shutoff function does not strongly depend on the full X-ORF sequence.

To investigate whether the effect of the FS mutation on host shutoff activity of UDL-01 segment three seen with plasmid-based assays could be recapitulated with infectious virus, mutant viruses were generated by reverse genetics. MDCK cells were then infected with WT and FS UDL-01 viruses at a high MOI [5], and pulsed with puromycin for 30 min to label nascent polypeptides [36], before being lysed. Lysates were then run on SDS-PAGE Western blotted for puromycin (Fig. 2c). The region of the blot corresponding to ~80–50 kDa, above where a pair of virally induced protein species (Fig. 2c, arrowheads) were seen, was assessed by densitometry to measure host protein synthesis levels within the cell. UDL-01 WT virus reduced cellular protein synthesis by over 50 % compared to uninfected cells while the FS mutant only caused <20 % host shutoff (Fig. 2d). Furthermore, we have recently shown that UDL-01 WT virus is able to cause host cell shutoff in avian cells [37]. These data corroborate the plasmid-based methods previously used and showed that in the context of infectious virus, UDL-01 expresses a classically active PA-X protein.

### PA-X expression does not affect H9N2 polymerase activity

Influenza PA-X has been suggested to modulate polymerase activity [15, 22, 24, 27], although these studies did not all agree on whether PA-X promotes or suppresses polymerase activity; the effect may be strain- as well as cell-type dependent. Therefore, we investigated the effect of PA-X expression on H9N2 polymerase activity in avian and mammalian cells. Cells were co-transfected with plasmids encoding the polymerase components and NP, alongside a viral RNA-like reporter encoding luciferase. In 293Ts, PB2 and PB1 from the mammalian adapted strain PR8 were used to overcome the restriction of avian IAV polymerase in these cells, whereas in avian DF-1 cells, the full polymerase from UDL-01 was used. Exchanging WT UDL-01 segment three with the different PA-X mutants had no significant effect on polymerase activity in either mammalian or avian cells (Fig. 2e, f). Furthermore, upon Western blotting cell lysates, no differences in PA or tubulin expression were seen, further confirming that PA-X expression did not alter PA accumulation (Fig. 2g).

### Viruses with abrogated PA-X expression have a minor replicative defect in mammalian but not avian cells

Although no difference in polymerase activity was seen between the different PA-X mutants, multicycle growth curves were performed in mammalian and avian systems to determine whether differences in PA-X may affect viral replication in a more biologically relevant context. PA-X removal has been shown to impact viral replication in several previous studies [15, 22, 23, 27], though similarly to polymerase activity these studies tend to disagree about whether PA-X expression enhances or suppresses viral replication and here too, the effect may be virus strain and host-specific.

**Fig. 3.**
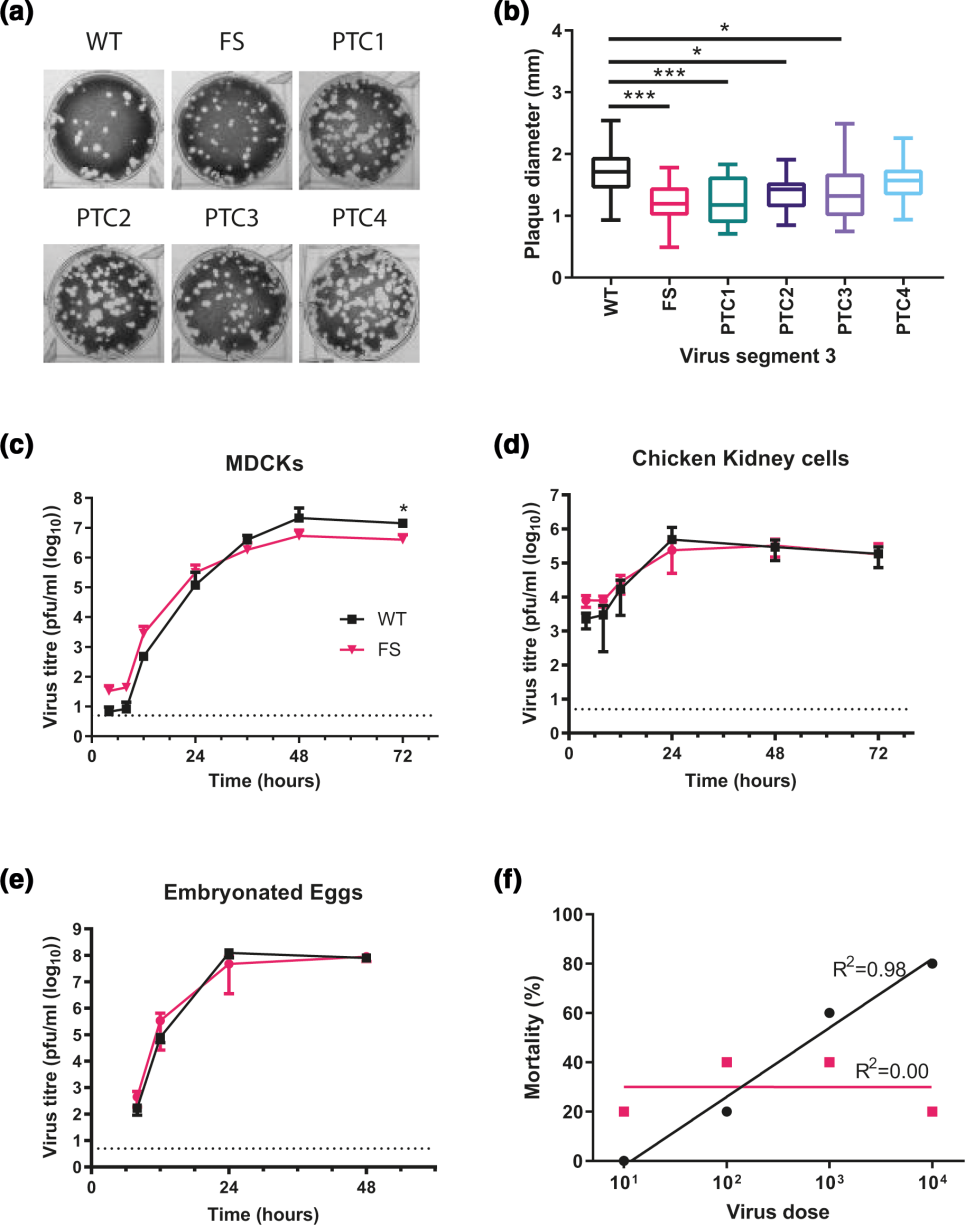
PA-X deficient viruses have a minor growth defect and lower *in ovo* mortality. (a, b) Viruses were rescued using reverse genetics and titrated under a 0.6 % agarose overlay in order to ascertain the plaque phenotype. (a) After 72 hours cells were fixed and stained with 0.1 % crystal violet solution and plates imaged. (b) ImageJ analysis software was used to measure the diameter of 20 plaques per virus. Graph represents average diameter of 20 plaques ±SD. Statistical significance was determined by one-way ANOVA with multiple comparisons. (c) MDCK cells or (d) CK cells were infected with a low MOI (0.01) of WT or FS H9N2 viruses. Cell supernatants were harvested at 4, 8, 12, 24, 36, 48 and 72 h post-infection and titrated via plaque assay. Graphs represent an average of three independent experiments ±SD. (e) The 10 day old fertilised hens’ eggs were infected with 100 p.f.u. of each virus. Allantoic fluid was collected at 4, 8, 12, 24 and 48 h post-infection and titrated via plaque assay. Graph represents an average of five eggs per virus per time point ±SD. Statistics through determined by Mann-Whitney U test. Dotted lines indicate a limit of detection of five plaques of the plaque assay. (f) Embryonated hens’ eggs were infected with different doses of virus, at 84 h post-infection, total embryo mortality after infection with the indicated viruses at each viral dose was calculated. Line represents non-linear fit of data with each data point represent % mortality at the viral dilution. *P* values for statistics throughout: *, 0.05 ≥P >0.01; ***, 0.001 ≥P >0.0001.

Initially, plaque size in MDCKs was assessed to determine if any gross replication defects could be seen in the mutants as plaque phenotype is a proxy for replicative fitness in influenza viruses. A modest, but statistically significant reduction in plaque diameter was observed within UDL-01 when PA-X was removed or truncated up to PTC3 (Fig. 3a, b). Average plaque diameters decreased from 1.7 mm to 1.2 mm, 1.25 mm, and 1.36 mm respectively. As UDL-01 FS had the largest impact on plaque diameter and host shutoff, replication kinetics of this virus were examined. MDCKs were infected at a low MOI and virus titres were assessed over a time course. UDL-01 WT and FS showed very similar growth kinetics, although UDL-01 WT displayed significantly higher titres at 72 h post-infection compared to UDL-01 FS (Fig. 3c). From 48 h post-infection there was a trend for decreased viral titres with UDL-01 FS compared to UDL-01 WT, implying in this particular H9N2 strain PA-X expression may slightly enhance viral replication in MDCK cells. However, when a similar growth kinetics experiment was performed in avian primary chicken kidney (CK) cells, there was no significant difference between UDL-01 WT and FS at any time point, suggesting a host-specific effect (Fig. 3d). To assess this further, replication kinetics were assessed in 10-day-old fertilised hens’ eggs. Eggs were inoculated with 100 p.f.u. of each virus and allantoic fluid was harvested periodically. *In ovo*, UDL-01 WT and FS viruses did not exhibit any significant differences in viral replication throughout the course of infection (Fig. 3e). Overall, the impact of mutating PA-X on the replication of UDL-01 was variable and the small differences appeared to be host-dependent, with PA-X playing a role in replication in mammalian, but not avian systems.

### Viruses with abrogated PA-X expression have lower embryonic lethality

PA-X expression has previously been shown to alter viral pathogenicity in animal models e.g. [11, 22–24, 27, 28] as well as *in ovo* [25]. Prior to performing an *in vivo* experiment we infected 10-day-old embryonated hens’ eggs with serial dilutions of UDL-01 WT or FS mutants and assessed embryonic lethality over 84 h, as previously described [25]. When percentage survival was plotted against viral dilution a clear difference could be seen between UDL-01 WT and FS (Fig. 3f). The inoculum size of UDL-01 WT negatively affected embryo survival in a dose dependant manner as seen by the ascending trend line (R^2^=0.98, ρ=1 by Spearman’s rank correlation coefficient), whereas UDL-01 FS survival was not dose-dependent (R^2^=0.00, ρ=0 by Spearman’s rank correlation coefficient). Overall, these data were suggestive of a difference in a pathogenicity between the WT and PA-X deficient viruses with reduction of PA-X expression potentially leading to lower pathogenicity *in ovo*, as we have previously described [25].

### Viruses lacking PA-X expression have delayed shedding and reduced visceral tropism *in vivo*

**Fig. 4.**
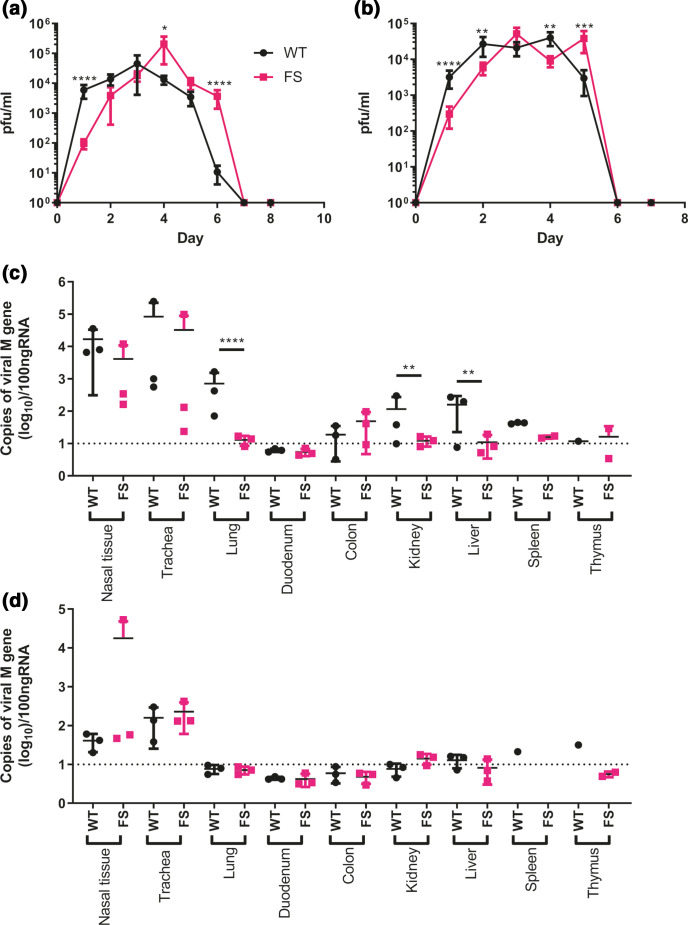
A lack of PA-X results in delayed shedding *in vivo* and restricted organ tropism. Birds were directly infected with either WT or FS H9N2 virus and naive contact birds were introduced 1 day post-inoculation. Swabs were taken from the buccal cavity throughout the study duration and virus titrated via plaque assay. The average ±SD buccal shedding profile of at least four birds per group are shown for (a) directly infected birds and (b) contact birds. Statistics determined by Mann-Whitney U Test. (c, d) Detection of viral M gene within tissues of (c) directly infected and (d) contact birds. On day 2 post-infection three birds per group were culled, a panel of tissues taken and following RNA extraction qRT-PCR for viral M gene carried out. Dotted lines indicate limit of detection. CT values were compared to a M gene standard curve to determine copy number. Graphs show mean RNA copy number ±SD. Statistics determined by unpaired *T*-test throughout. *P* values for statistics throughout: *, 0.05 ≥P >0.01; **, 0.01 ≥P >0.001; ***, 0.001 ≥P >0.0001; ****, *P* ≤0.0001.

As the effect of PA-X expression by H9N2 viruses has yet to be assessed in their natural host, we performed an *in vivo* experiment to test the effect of PA-X on virus replication, transmission, tropism, pathogenicity and cytokine expression in chickens. Groups of ten 3 week-old White Leghorn (VALO breed) chickens were inoculated intranasally with 10^4^ p.f.u. of either UDL-01 WT or FS virus (or sterile PBS). One day post-inoculation, eight naïve contact birds were introduced into each directly infected group to assess viral transmission. Birds were swabbed daily in both buccal and cloacal cavities to determine viral shedding. Throughout the study period birds were monitored for clinical signs, however only very minimal signs were seen in any birds (data not shown). Furthermore, in both directly infected and contact birds, no culturable virus was detected from cloacal swabs. Directly infected birds in both groups showed robust buccal shedding from days 1–6, peaking at titres of over 10^4^ p.f.u. ml^−1^ (Fig. 4a). However, birds infected with UDL-01 FS showed a delayed buccal shedding profile compared to UDL-01 WT infected birds, shedding significantly less on days 1–2. By day 3 post-infection, shedding levels were comparable between groups and by day 6, an increased number of animals infected with UDL-01 FS shed compared to WT (2 of 7 for UDL-01 WT vs 4 of 6 for UDL FS). Within both groups, viral shedding was cleared by day 7 post-infection. To estimate the total amount of virus shed by each group, the area under the shedding curves (AUC) were calculated for both viruses; giving 81 674 for WT UDL-01 versus 241 229 for the FS mutant. This suggested that UDL-01 FS infected birds shed more virus buccally over the course of the experiment than the UDL-01 WT infected birds.

All contact birds in both groups became infected and showed buccal shedding from 1 day post-exposure to the directly infected birds, indicating robust contact transmission of both viruses (Fig. 4b). A similar buccal shedding pattern to the directly infected birds was seen in the contact birds; UDL-01 FS again showed delayed shedding kinetics, with significantly less virus shed on days 1–2 post-exposure and significantly more virus shed by day 5 post-exposure. Within both contact groups viral shedding was cleared by day 6 post-exposure. When AUC values were calculated for the contact bird populations, values were comparable; UDL-01 WT AUC was 94 171 whereas the UDL-01 FS AUC was 104 655. Overall, the shedding profiles of the infected birds suggested that expression of PA-X by the WT virus led to accelerated, but not increased, buccal shedding of virus compared to a virus which lacked PA-X expression.

On day 2 post-inoculation three birds from each group – directly infected, contact or mock – were euthanised and a panel of tissues were taken to assess viral tropism and cytokine profiles. After RNA extraction, qRT-PCR for the viral M gene was performed to assess viral replication within different tissues. In tissues isolated from directly infected birds, viral replication was primarily observed within the upper respiratory tract in both UDL-01 WT and UDL-01 FS infected birds (Fig. 4c). There were no significant differences in M gene copy number within the nasal tissue and trachea. However, significantly more viral RNA was detected for UDL-01 WT in the lower respiratory tract in the lung. Within other visceral organs little viral RNA was detected, particularly in tissues collected from UDL-01 FS infected birds. UDL-01 WT RNA could be detected in both the liver and kidneys to significantly higher levels than UDL-01 FS. Therefore, UDL-01 WT virus showed increased viral dissemination compared to UDL-01 FS at day 2 post-inoculation. Tissues taken from the contact birds at day 2 of the experiment (i.e. day 1 post-exposure), again showed robust RNA levels in the upper respiratory tract tissues, the nasal tissue and trachea (Fig. 4d). Viral loads were not significantly different between UDL-01 WT and FS infected birds in any tissues and very low levels of RNA were detected in non-respiratory tissues. Overall, these data show that removal of PA-X from UDL-01 led to reduced viral dissemination at day 2 post-infection for directly inoculated birds.

### Cytokine expression in the upper respiratory tract of directly infected birds correlated with viral titres

PA-X is known to alter host responses, at least in part by downregulating protein synthesis [11]. Within different host species, modulation of PA-X expression has been shown to alter host expression of cytokines and chemokines; for example an H9N2 AIV unable to express PA-X has been shown to have decreased expression of *IL-6*, *IL-1β*, *CCL3*, *IFN-γ* and *TNF-α* within a mouse model compared to a virus with PA-X expression [23]. Therefore, it was assessed whether UDL-01 WT and FS led to differential cytokine and chemokine responses within the chicken host. The upper respiratory tract tissues were chosen due to robust and comparable viral replication in directly infected animals (Fig. 4c). qRT-PCR for a range of chicken cytokines and markers of the interferon response were assessed. The host gene, *RPLPO-1*, was used for gene normalisation.

**Fig. 5.**
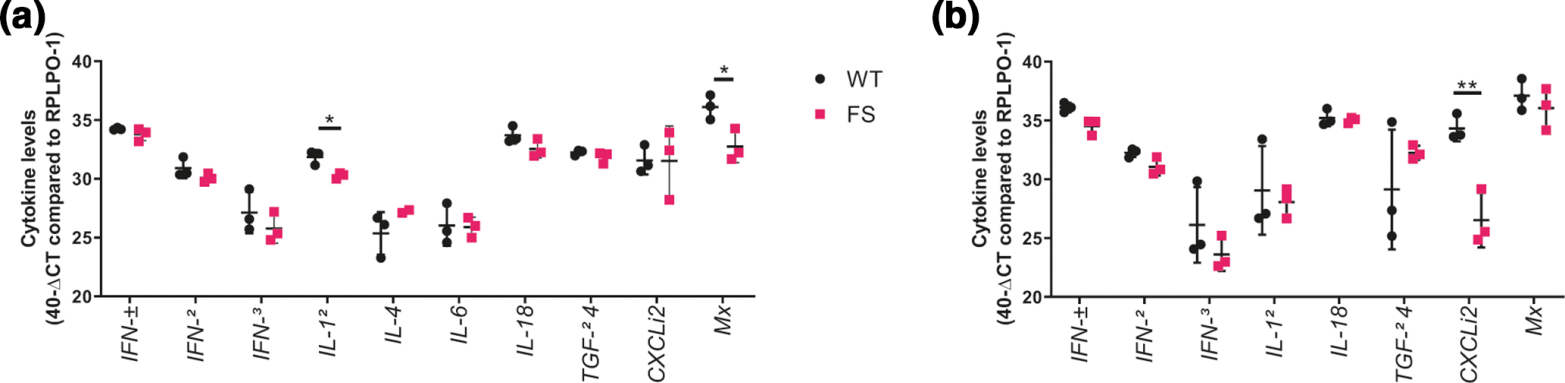
Cytokine induction in H9N2 infected birds. RNA was extracted from nasal tissue (a) or trachea (b) of WT and FS infected chickens. qRT-PCR was performed for a panel of cytokines and levels compared to the reference gene, *RPLPO-1* were calculated. Cytokine expression for each bird is represented in a single point, with error bars displaying mean ±SD of tissues from *n*=3 chickens. Statistics were determined by Unpaired *T*-tests. *P* values for statistics throughout: *, 0.05 ≥P >0.01; **, 0.01 ≥P >0.001.

Nasal tissue from directly infected birds displayed little differences in immune response (Fig. 5a). Some minor differences were seen with expression of *IFN-β*, *IFN-γ* and *IL-18* with UDL-01 WT infected birds generally expressing higher levels of cytokines. These differences only reached significance with the expression of *IL-1β* and the innate immune effector gene *Mx*, with UDL-01 WT infected birds expressing higher levels of these immune markers. Immune responses in the tracheas of directly infected birds showed a similar trend to those in the nasal tissue (Fig. 5b). Few differences in cytokine expression were seen between UDL-01 WT and UDL-01 FS infected animals. UDL-01 WT infected animals again tended to have increased expression of *IFN-β*, *IFN-γ* and *IL-1β* although this only reached significantly different levels with *CXCLi2*. It was worth noting that although UDL-01 WT trended towards having higher levels of cytokines in the upper respiratory tract, these tissues also had higher levels of viral RNA, therefore it is difficult to draw conclusions about whether PA-X is having a direct role on cytokine expression; the trend for reduced cytokines in FS mutants may be a result of reduced viral RNA in tissues infected with these viruses.

## Discussion

In this study we set out to investigate whether PA-X expression is a virulence factor for H9N2 viruses in their natural poultry host. As expected, ablating PA-X expression resulted in loss of host shutoff activity, showing that, as with other IAV strains, the shutoff activity of H9N2 viruses is partly due to PA-X expression. We found that PA-X expression resulted in slightly increased replication in mammalian MDCK cells but had no effect on titres in primary chicken cells or eggs, although virus with PA-X caused more embryonic lethality *in ovo* at higher input doses. We further found that *in vivo*, ablating PA-X expression led to a delayed shedding profile and lower visceral organ tropism. These results, the first study to investigate the impact of PA-X expression in a low pathogenicity avian influenza virus in its natural host suggest that PA-X expression aids faster virus replication and dissemination *in vivo*, although we saw little evidence for this acting through viral modulation of induced cytokine levels.

The finding here that expression of PA-X causes more rapid virus shedding after infection but that a virus with ablated PA-X expression potentially sheds for longer than WT virus is similar to what has been seen for H1N1-infected mice [15] and H9N2-infected mice [23]. The latter study showed that when mice were infected with a different lineage of H9N2 virus to the one used here (BJ94 versus G1) that had PA-X ablated, there was decreased virulence associated with reduced virus titres in mouse lungs. Interestingly, PA-X tells a contrasting story in high pathogenicity avian influenza viruses and the 1918 H1N1 pandemic virus. Loss of PA-X in 1918 H1N1 and HPAI H5N1 viruses caused increased virulence in mice [11, 21–23] and in ducks and chickens [27]. While Jagger and colleagues did not link the increased virulence in mice of 1918 H1N1 IAV lacking PA-X to effects on virus replication, Gao and colleagues showed that increased virulence in mice after loss of H5N1 PA-X was associated with increased titres of the PA-X null virus in the lungs, brain and blood of infected mice. Similarly, Hu and colleagues showed that increased virulence in chickens, ducks and mice on loss of H5N1 PA-X correlated with increased virus titres of the PA-X null virus.

The authors of the H9N2 infection study in mice (Gao *et al*. 2015c) proposed that differences in effects of PA-X on virulence could be due to the fact that high pathogenicity viruses induce high levels of cytokine responses but low pathogenicity viruses do not typically induce high levels of cytokines. Since a ΔPA-X mutant of a low pathogenicity virus is less effective at host cell shut off, it was more effective at eliciting an antiviral response resulting in reduced virus replication in their study.

Although no difference in pathogenicity in viruses expressing or lacking PA-X was seen in this study it should not be ruled out there may be a role. UDL-01 WT has shown to be a good model for a moderately pathogenic H9N2 virus in previous studies, causing clear clinical signs and even limited mortality [38, 39]. However these studies used Rhode Island Red breed chickens, whereas here we used white Leghorn birds which appear to be more resilient to avian influenza virus infection [40, 41]. Therefore, it is possible that the virus lacking PA-X is less pathogenic than UDL-01 WT, but this was not observed due to the lack of clinical signs and mortality in the UDL-01 WT infected groups in this system. It is worth noting we have previously correlated visceral tropism with pathogenicity and clinical signs in UDL-01 [38, 39]; in this study UDL-01 WT did show greater visceral tropism than UDL-01 FS, perhaps suggesting an attenuated pathogenicity when PA-X expression is ablated.

Overall, this work suggests PA-X may play a role in H9N2 viruses in birds by allowing more rapid replication and dissemination throughout the host, potentially leading to higher pathogenicity. This work will be useful in future surveillance efforts allowing the assessment of newly sequenced viruses as it suggests viruses expressing a full-length PA-X are likely to have a wider tropism and higher pathogenicity than those that do not. Furthermore, this work suggests that there may be slightly different roles for PA-X in mammalian and avian hosts, potentially helping explain the mechanism by which PA-X works.
